# The Emotional States Elicited in a Human Tower Performance: Case Study

**DOI:** 10.3389/fpsyg.2021.611279

**Published:** 2021-04-01

**Authors:** Sabrine Damian-Silva, Carles Feixa, Queralt Prat, Rafael Luchoro-Parrilla, Miguel Pic, Aaron Rillo-Albert, Unai Sáez de Ocáriz, Antoni Costes, Pere Lavega-Burgués

**Affiliations:** ^1^Motor Action Research Group (GIAM), INDEST, National Institute of Physical Education of Catalonia (INEFC), University of Lleida (UdL), Lleida, Spain; ^2^Research Group on Youth, Society and Communication (JOVIScom), Pompeu Fabra University, Barcelona, Spain; ^3^Motor Action Research Group (GIAM), Institute of Sport, Tourism, and Service, South Ural State University, Chelyabinsk, Russia; ^4^Motor Action Research Group (GIAM), INDEST, National Institute of Physical Education of Catalonia (INEFC), University of Barcelona (UB), Barcelona, Spain

**Keywords:** ethnomotricity, intangible cultural heritage, traditional sporting game, motor praxiology, human towers

## Abstract

Human Towers are one of the most representative traditional sporting games in Catalonia, recognized in 2010 as Intangible Cultural Heritage by the United Nations Organization for Education, Science and Culture (UNESCO). The objective of this research was to study the emotional states (well-being, discomfort, and affectivity) elicited by a representative performance of the *colla* de Castellers de Lleida. This research is based on an ethnographic case study, with mixed methods in which 17 key informants (*castellers*) voluntarily participated. Participant observation was used; the data were recorded in a field diary and oral sources (semi-structured interviews). The content analysis was done using the Atlas.ti software (version 8.4.4). An SPSS database was also created. The statistical techniques were: Descriptive statistical techniques, cross tables with Pearson's Chi-square values (significance level of *p* < 0.05). We also used a classification and regression trees (CRT) to examine the predictive capacity of five independent variables (data source, logic, semantic units; contexts of a performance) of emotional states. The results reveal that the comments (*n* = 132) were mostly oriented toward well-being states (*n* = 70; 53%), The internal cooperative logic of the Human Towers enhances the intense interpersonal relationships of socio-emotional well-being.

## Introduction

The European ludic tradition has provided an extraordinary variety of ways of dialogue and relationships among citizens. Traditional Sporting Games (TSG) are a mirror of the social, linguistic, and cultural richness of the different territories and they become intangible cultural heritage (ICH). UNESCO (2003) states that TSG are part of the ICH and are a symbol of the cultural diversity of our societies.

The Human Towers (HT) is a ludic practice that is deeply rooted in the territory of Catalonia (Spain) where it has historically been present in the festivities of Catalan towns and cities. To build a HT requires the motor collaboration of a group of participants that can exceed several hundred people. The socio-motor and cooperative nature of the HT (Parlebas, 2001) fosters fundamental skills for today's society: teamwork, commitment to the collective, tolerance, solidarity, and the spirit of self-improvement (Coordinadora de Colles Castelleres de Catalunya, 2012). These universal human values allowed UNESCO to recognize HT as Intangible Cultural Heritage (ICH) on 16 November 2010 (UNESCO, 2010).

This article aims to identify the castellers as a community or micro-society (Parlebas, 2001) that, by participating in the construction of HT, elicites different emotional experiences among its members. Accordingly, it will be possible to verify that this community of social relationships is also an emotional community (Costes et al., 2021).

### Playing a Game Means Learning to Live Together and to Feel Emotions

There is currently scientific consensus on understanding emotions as biological states of the nervous There is currently scientific consensus on understanding emotions as biological states of the nervous system brought on by neurophysiological changes variously associated with thoughts, feelings, behavioral responses, and a degree of pleasure or displeasure (e.g., Panksepp, 1982; LeDoux and Hirst, 1986; Ekman, 1992; Damasio, 1998).

From this point of view, players who participates in a game or sport not only has to overcome challenge of motor nature, but also engages in an emotional goal in order to manage their emotions intelligently (Lavega et al., 2014). Poor management of their emotions (e.g., feeling excessive fear, when the construction of a Human Tower is unbalanced) can distort the appreciation of the distance, duration, or difficulty of the task. However, carrying out a task with pleasure can lead the players to develop their motor and affective potentialities (Parlebas, 2001).

For the sociologist Kemper (1981), emotion depends on the interpretation of the situation in which it is expressed. When a situation appears as a relevant stimulus for the players, it brings them well-being and therefore satisfy their expectations. Then the actors feel positive emotions such as joy. If, on the other hand, the situation causes discomfort and does not fulfill their expectations, the emotion that arises is negative, such as fear, anger, rejection, or sadness (Bisquerra, 2003; Lavega et al., 2018).

Collins (2009) indicates that the exchange of “emotional energy” in social interactions facilitates the emergence of social structures. According to this view, TSG could act on an exchange of relationships and also of emotions, based on giving and receiving affection in which the protagonists share successes and failures, positive and negative emotions, rewards and deprivations, prizes, and punishments.

Physical activity professionals have an extraordinary tool for interpreting the social construction of emotions: TSG. This is a legacy that can be observed in any society whose relationship structures favor a social construction of emotions based on features of local culture (Lavega et al., 2016).

In the process of learning to be social beings, the TSG has a fundamental role. By participating in TSG, the person discovers the pleasure of living with others, the search to “live together,” to communicate and to share common emotions according to features of the local culture. The TSGs act as extraordinary ecological mechanisms (Milton, 2005) in the “emotional alphabetization” of their people. By playing we are constantly accumulating affective experiences in which coexistence with others is essential in the process of socializing the cultural guidelines established by a community. Each person generates their own emotions and experiences in two directions while playing: on the one hand, toward themselves, bearing witness to the subjective process of this experience, and on the other hand, building a network of emotions shared and complemented by the other participants, which fosters the learning of ways to dialogue and to feel emotions regarding significant social events (Keltner and Haidt, 1999).

### To Study the Emotions Originated in the TSG Under the Light of Ethnomotricity

To interpret the existing relationship of the distinctive features of the game (internal logic) with the local culture (external logic), motor praxeology incorporates the concept of ethnomotricity (Parlebas, 2001) referred to “the field and nature of motor practices, considered from the point of view of their relationship with the culture and social environment in which they have been developed” (p. 227).

All TSG has an internal logic, an identity card that provides specificity, since it triggers an internal order that requires participants to adapt to a way of relating to other participants, with space, with material and with time (Parlebas, 2003). The internal logic of any TSG corresponds to the system of obligations imposed by the rules, or convention of any motor situation. That is why, “despite appearances, playful behaviors are not anarchic, but are strongly determined by the reason of the rules” (Parlebas, 2002, p. 147).

The internal logic of games is an excellent mirror to observe the set of social-emotional relationships and learning that activate the TSG. Recognizing the connection of the TSG with the local culture means considering that the internal logic of a TSG can be reinterpreted from the outside, by an external logic associated with sociocultural conditions that attributes new, unusual, or specific symbolic meanings to it (Parlebas, 2003). Internal logic focuses on the study of the properties of four types of internal relationships based on the rules of a game (Lagardera et al., 2018): the type of motor interaction among the participants (in the HT everyone performs as playmates and they intervene with the same rights and prohibitions); the relationship with space (depending on the type of human tower there will be a certain number of floors and people on each floor); the relationship with time (in the HT there are two main phases of loading and unloading the human tower); the relationship with the material (in the HT the use of the *gralla*^1^ and the use of the sash that helps the *castellers*^2^ to perform their HT ascending actions.

**Figure 1:**
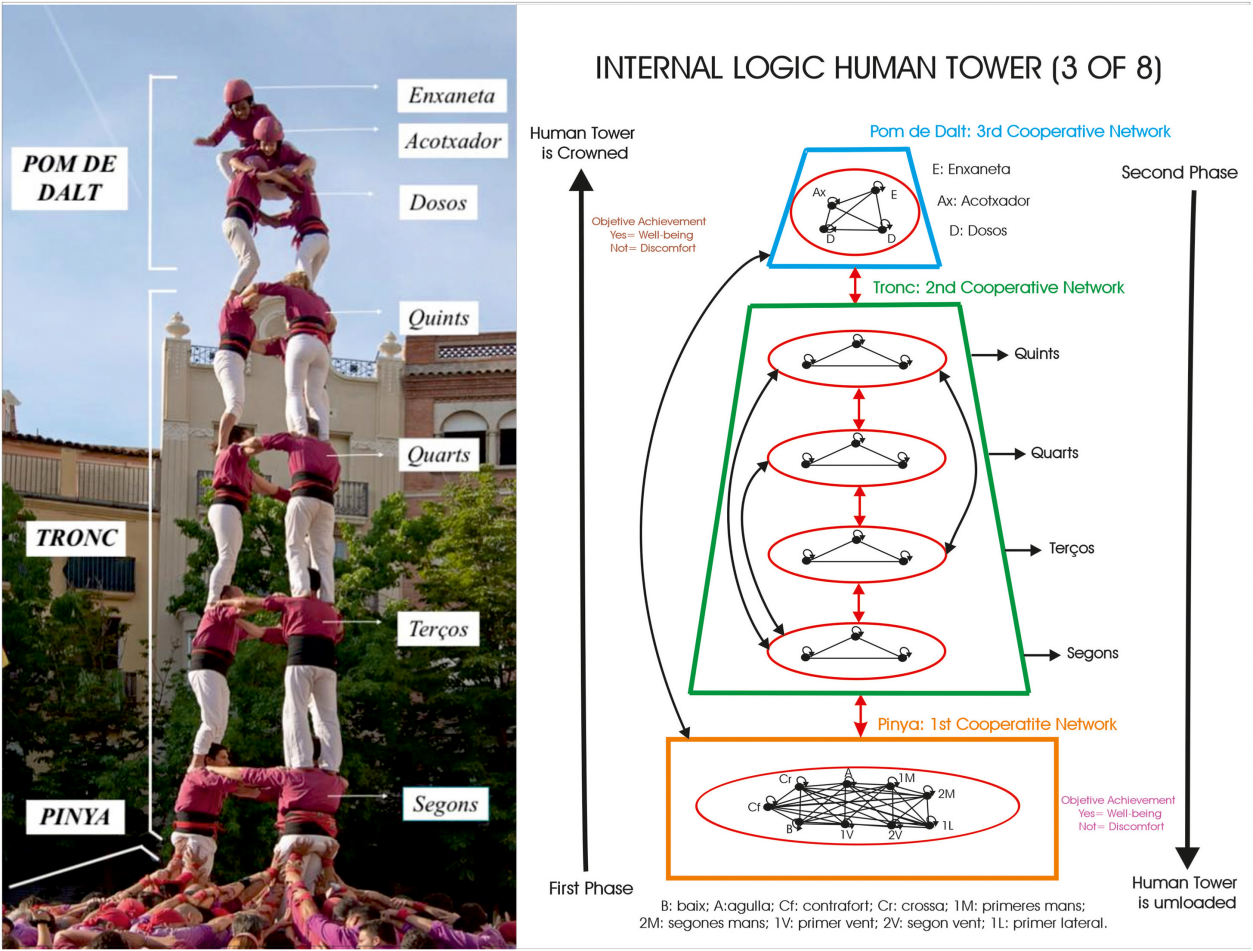
Parts of a human tower three of eight (photo author's own) and its corresponding motor communication network associated with emotional states. On the right, graph theory is applied to represent the *castellers* (points) and their cooperative communication relationship (lines) between people on the same floor and also on different floors, in the phase of loading, unloading. All this provokes emotional states of well-being or discomfort depending on the result of the performance.

The internal logic of the HT is constituted by a network of cooperative motor communication (Parlebas, 2001) associated with a set of positive and interdependent interactions among the people on the different floors or motor communication sub-networks (MCN): The *castellers* who are on *the pinya*^3^ (1st MCN) build the solid base of the human tower; *the tronc*^4^ (2nd MCN) and the *pom de dalt*^5^ (3rd MCN) collaborate doing the construction on the vertical in order to achieve the common goal of loading and unloading the human tower (Costes and Lavega-Burgués, 2019) (Figure 1).

The link between the game and the local socio-cultural context or external logic can be revealed through the characteristic traits of the conditions of practice (Lagardera et al., 2018): the characteristics of the protagonists: age and sex (in HT people of different ages and sex participate in all floors: in the *pinya* the *castellers* are adults and elderly people; in the *tronc* the protagonists are adult people; and in the *pom de dalt* only children are involved); playing areas (HT are built in the square and also in the training center); moments (HT usually follow a festive calendar); materials (each *colla*^6^ wears clothes and materials with colors that identify them as a singular community). At the same time, the external logic also refers to the values and meanings given by each person or social group, for example, the emotional and relational meaning that the *castellers* give to the experience of being a *casteller* and building HT.

From this ethnomotor perspective, when building a human tower, the participants adapt to the internal logic of this practice, in a contextualized way, i.e., in accordance with the features of the local culture of their community or external logic. In each of the moments that make up the experience of being a *casteller* (e.g., in the training and in the performances in the square), each *casteller* experiences emotions (Panksepp, 1982; Ekman, 1992; Levenson, 1992; Izard, 1994). These are momentary experiences, emotional states associated with each moment, which gradually weave an affective imprint on the actors (Kemper, 1981; Lavega et al., 2016, 2018). In other words, the HT construct emotional meanings from a subjective point of view, but also collectively in accordance with their social norms. This union of emotion with local culture allows us to affirm that the social norms of the HT are also emotional norms for that community (Hochschild, 1979). For all these reasons, it seems reasonable to affirm that the practice of HT favors the social construction of emotions.

Taking the theoretical reference framework as a starting point, this research aimed to study the emotional states experienced by the group of Castellers de Lleida at different stages of the construction of the human towers in the main square of the city of Lleida, the Paeria Square.

## Method

### Design

This research corresponded to an ethnographic case study (Martins, 2006; Hernández et al., 2014). Regarding the type of analysis, it is a mixed methods investigation (Anguera et al., 2014; Lagardera et al., 2018) since the statistical analysis of quantitative data is combined with the qualitative analysis of narrative data.

### Participants

Seventeen key informants participated voluntarily: 16 *castellers* representing the main positions in the human tower modality called three of *eight* (a building composed of three pillars and eight floors). Among these informants, 8 people (5 men and 3 women, age range 34–67 years) were representatives of positions on the first floor of the human tower, called *pinya*; 5 informants (3 men and 2 women, age range 22–46 years) were representatives one from each floor of the *tronc*; and 3 informants were representatives one from each floor of the *pom de dalt*, respectively, from the floor of *dosos* (boy who was 11 years old), *acotxador* (girl who was 10 years old) and e*nxaneta* (girl who was 8 years old). The other key informant was 1 person (man, 30 years old) who had had the institutional function of *cap de colla*^7^ in the past. All gave informed consent to participate in this study. In addition, this research was approved by the Clinical Research Ethics Committee of the Sports Administration of Catalonia (08/2019/CEICEGC).

### Procedures

Two ethnographic strategies or procedures were used to obtain data:

#### Participant Observation

In this procedure, the researcher in the fieldwork combined the role of observer and participant as a *castellera* of the *colla*. This process was developed systematically for 3 years in the *colla* de Castellers de Lleida, following 9 performances of HT in Paeria Square in Lleida the context on which we focused our attention in this study.

**(a) Observer**. This was the first phase of observation of the participation of the members of the group in a performance in Paeria Square, the notes were taken at the same time as they happened.
**(b) Participant**. In the second phase, as a *castellera* participant, the researcher took notes of her participation in the performances in Paeria Square after the end of each performance.

#### Oral Sources

Semi-structured individual interviews were carried out in the university premises where the study was carried out. The script of the interviews was linked to the position of the *castellers* in the human tower, in this case we chose a specific modality, the human tower three of eight as a reference for all the interviews.

### Instruments

#### The Field Diary

The notes from the participant observation were recorded in a field diary. Narratives referring to information on a day's performance in Paeria Square were noted down; the stages of the performance were described; the types of human towers that were performed; the use of the space once in the square; the relationship between *castellers* and spectators, the expressions and comments that the *castellers* made during the performance with the researcher. In the second phase as a *casteller* participant, the researcher wrote down ethnographic information, describing in first person what happened during the performance, the use of space, the relationship between players, the perception of the spectators, the emotional states she felt at each moment, among other elements.

#### The Semi-Structured Interviews

The script of the semi-structured interviews was linked to the position of the *castellers* in human tower three of eight (Table 1). The interviews sought to gather information about their intervention in each of the sequences or situations in which they intervened (before, during, and after the construction of the human tower three of eight). These interviews were conducted by the researcher himself, who audio-recorded them with the permission of the participants and later transcribed them textually.

**Table 1:** Codes of oral sources (participants) and their positions in HT that are used in this manuscript.

**Code**	**Meaning**
E.1	Semi-structured interview (SI) *casteller*—*agulla* position
E.2	SI *casteller*—*enxaneta* position
E.3	SI *casteller*—*contrafort* position
E.4	SI *casteller*—*primeres mans* position
E.5	SI *casteller*—*quart* position
E.6	SI *casteller*—*segon vent* position
E.7	SI *casteller*—*baix* position
E.8	SI *casteller*—*quint* position
E.9	SI *casteller*—*segones mans* position
E.10	SI *casteller*—*crossa* position
E.11	SI *casteller*—*dosos* position
E.12	SI *casteller*—*acotxador* position
E.13	SI *casteller*—*terç* position
E.14	SI *casteller*—*primer lateral* position
E.15	SI *casteller*—*primer vent* position
E.16	SI *casteller*—*segon* position
E.17	SI *casteller*—*ex cap de colla*
N.I.	Researcher's notes on participant observation

### Data Analysis

A narrative database was created that allowed the content analysis of the field diary and the interviews to be carried out using Atlas.ti software (version 8.4.4) in the same hermeneutic unit.

The semantic units of meaning of the interviews and the field diary were identified, associated with different situations of the internal logic of the construction of a Human Tower (preparation, *pinya, tronc*, coronation, going down). Different contexts of the external logic of the HT were also analyzed (from the social premises to the plaza, celebration of the HT). To analyse the emotional states of well-being and discomfort, we followed the procedure indicated by Lagardera et al. (2018) on qualitative methodology in the study of traditional play. To identify the basic emotions, we followed the biopsychological models of Panksepp (1982); Ekman (1992); Levenson (1992), and Izard (1994), which currently have the most empirical support (Lavega et al., 2018). These models identify five basic emotions: one positive emotion, joy; and four negative emotions (anger, sadness, fear, and rejection).

Subsequently, a SPSS database was developed where: (a) descriptive statistical techniques were used; (b) cross tables with Pearson's Chi-square values, starting from the significance level of *p* < 0.05. Special attention was directed to adjusted residuals (AR) > 1.96 or < −1.96; and (c) classification and regression trees (CRT) (Liu et al., 2016) to examine the predictive capacity of five independent variables (data source; logic; semantic units; contexts of a performance) of the emotional states of the *castellers* performance. The dependent variable corresponded to comments oriented toward the expression of emotional states. When the orientation was directed toward reflecting positive emotions it was categorized as well-being (e.g., you feel very happy when you stand in your position), when the orientation was oriented toward negative emotions it was associated with discomfort (e.g., if the castle falls you feel frustration and anger). Finally, when the comments had a neutral emotional orientation, i.e., no clear orientation toward positive or negative emotions, it was called the category of affectivity (e.g., in Paeria Square it is twice as emotional as elsewhere). The multivariate technique (CRT) divides the nodes dichotomously, allowing the data to be explored and modeled appropriately (De'ath and Fabricius, 2000). The Gini impurity measure was applied (Thornton et al., 2016), and cross validation was implemented. The statistical analysis was performed with the software package SPSS version 24.0 (SPSS Inc., Chicago, IL, USA).

This study provides an original contribution by combining a qualitative and a quantitative analysis. The content analysis together with different statistical tests allows a better understanding of the interpretation of the study of the emotional states experienced by the *castellers*.

## Results

The Human Tower performance in Paeria Square originated 132 comments mainly oriented toward states of well-being (*n* = 70; 53%), followed by discomfort *(n* = 49; 37.1%) and affectivity in general (*n* = 13; 9.8%). Positive emotions were mainly triggered when the performance of the 3d8 HT was successful both in the phase of loading and unloading this human tower (e.g.,: “and when you go down you hug everyone because you feel very happy to be able to perform a 3d8 human tower” E.8; “when the 3d8 finishes you feel very happy, satisfied, very proud” E.5). Negative emotions were present when the *castellers* were not able to achieve one of their goals (e.g.,: “when the human tower falls you feel angry” E.13; “when we are unable to carry the human tower you feel very frustrated” E.1). On other cases, the *castellers* expressed an emotional engagement when participating in the building of 3d8 HT; however, these comments were not always oriented toward positive or negative emotions, which is why we considered data referring to affectivity in general (e.g.,: “in Paeria Square you feel more emotions than in other squares” E.6; “When I was not the leader of the *colla* I felt the human tower in a different way” E.4).

### The Emotional States Raised in the Contexts of a Performance in Paeria Square

**Figure 2:**
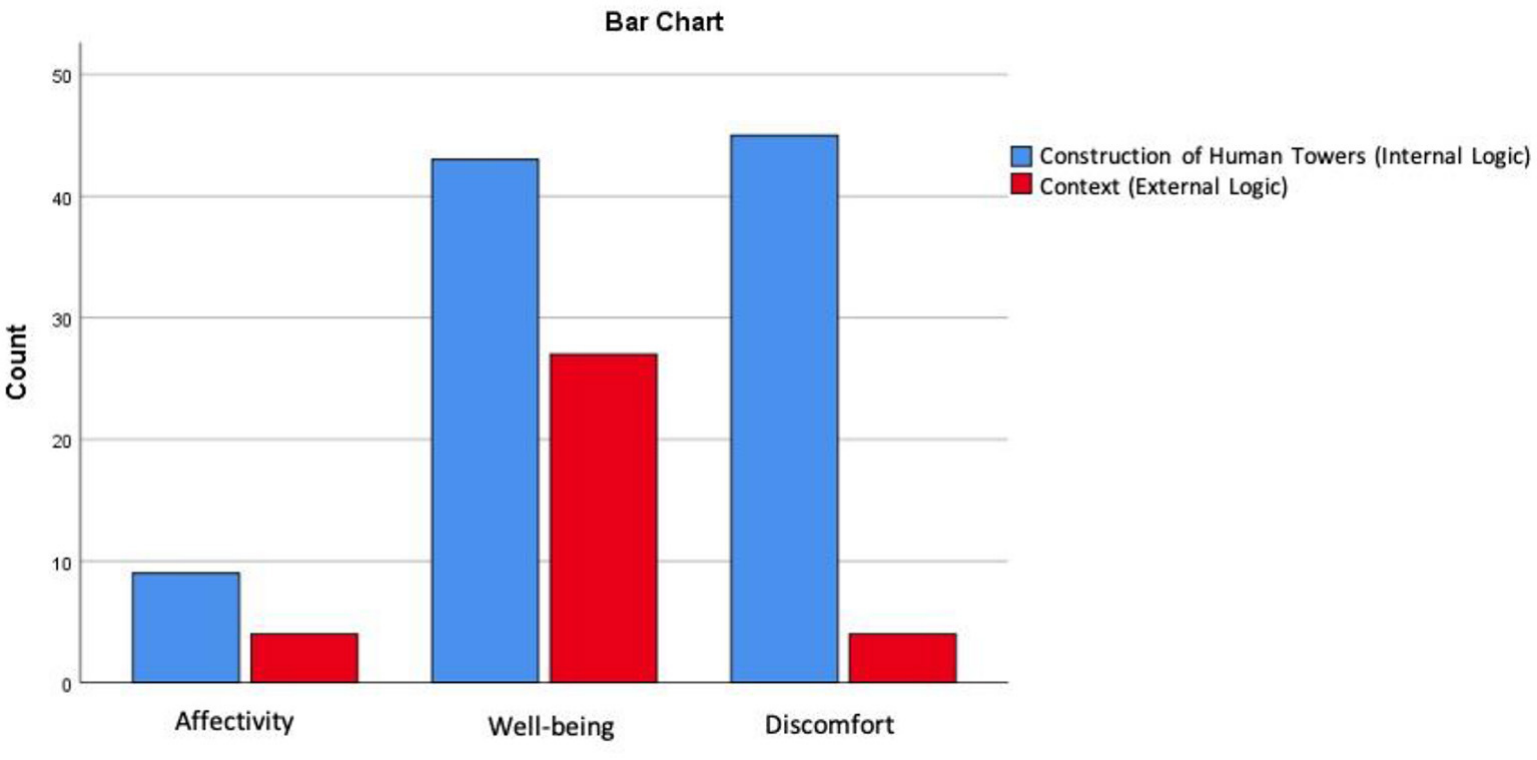
Emotional states originated by human towers.

The results showed that the description of the emotional states were originated mainly from the construction of the HT (internal logic) (*n* = 97; 73.5%) (Figure 2). Cross-tables indicate that HT significantly (*p* < 0.001) elicited states of discomfort (*n* = 45; *AR* = 3.7; 34.1%) and well-being (*n* = 43; *AR* = −3.3; 32.6%) although in the opposite direction.

### Predictive Capacity of Ethnomotor Variables on Emotional States

**Figure 3:**
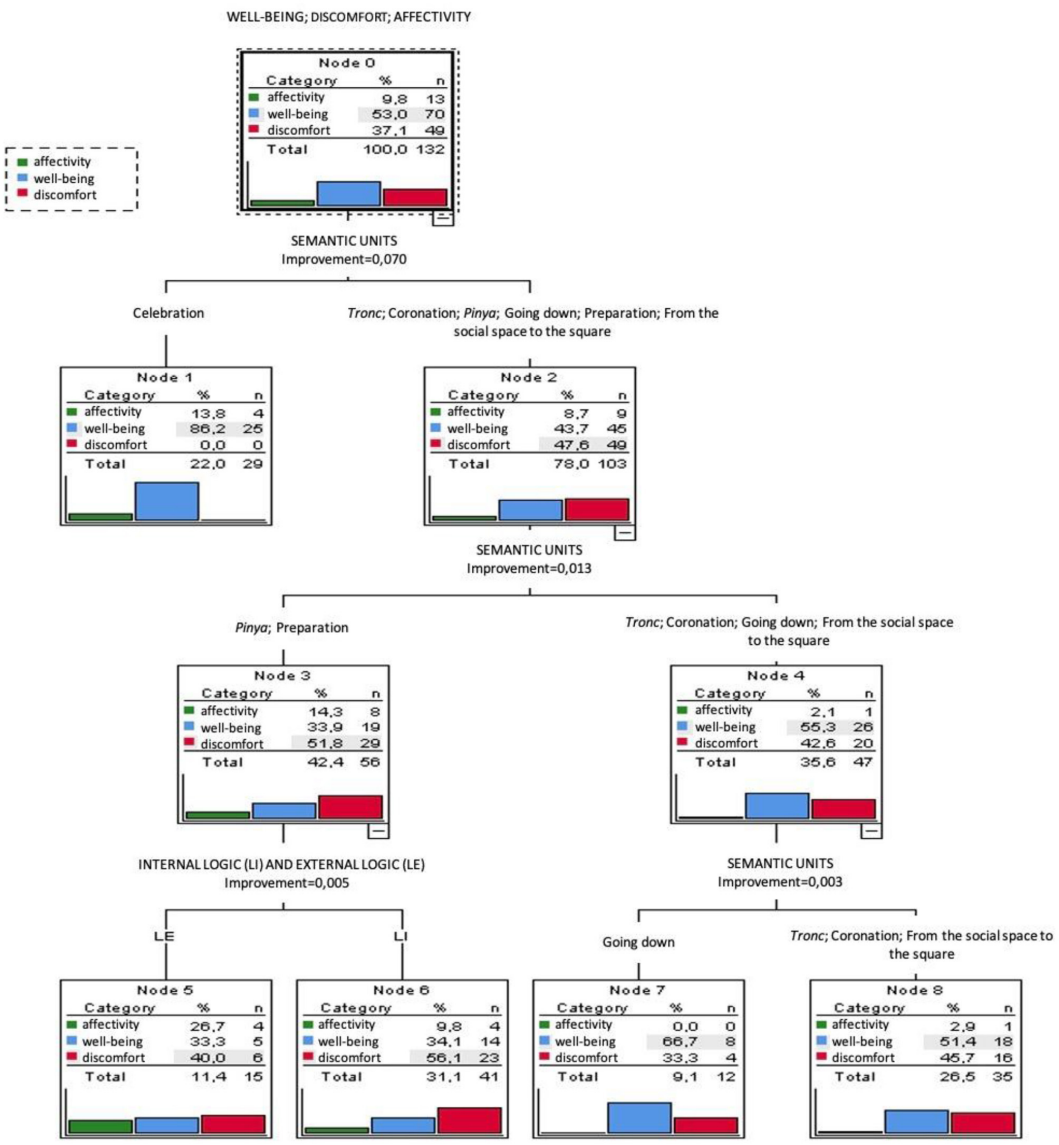
Classification and regression tree (CRT): predictive variables of emotional states.

The CRT only identified 2 predictive variables: semantic units; and logics (internal logic and external logic) (Figure 3). The result correctly classified 60.6% of the categories well-being, discomfort and affectivity.

The first predictive variable was the semantic units with significant differences (improvement = 0.070) between the celebration, with a predominance of well-being (*n* = 25; 86.2%) and absence of discomfort, and the rest of units with a slight predominance of discomfort (*n* = 49; 47.6%) compared to well-being (*n* = 45; 43.7%). At the second level, the tree found significant differences (improvement = 0.013), between node 3, *pinya* units and square preparation, and node 4, consisting of the rest of units. In node 3, comments of discomfort (*n* = 29; 51.8%) and well-being (*n* = 19; 33.9%) predominated. This trend is reproduced in a similar way at nodes 5 and 6 of the next level of the tree.

Node 4 had an inverse behavior, as well-being (*n* = 26; 55.3%) predominated over discomfort (*n* = 20; 42.6%). This behavior was similar in node 8 and was emphasized in node 7 represented by the unload the HT unit where well-being clearly predominates over discomfort.

## Discussion

The aim of this research was to study the emotional states experienced by the group of Castellers de Lleida at different stages of the construction of the HT in the main square of the city of Lleida, the Paeria Square. This is the main context in which this *casteller* community performs.

Statistical analyses of the comments revealed that the construction of a Human Tower (internal logic) is the unit that gives rise to the greatest number and variety of emotional states.

### The Internal Logic of the Human Towers. The Cooperative Network Activates Collective Emotional States

By cooperating in the construction of a HT, each *colla* puts into action a HT playful community that behaves like a micro-society (Parlebas, 2001) constituted by an intertwined union of the different floors. So that each floor constitutes an emotional network that is also part of another network or emotional floor. Cooperating and sharing emotions are two sides of the same phenomenon that elevate the *castellers* to a network of cooperative and also emotional networks.

Of course, in Paeria Square you feel much more nervous, you are already nervous from the beginning. (E.6)

The analysis of the comments reveals that the construction of a Human Tower triggers nerves and tension. These negative emotions are not always in fact disruptive feelings, since they help the *castellers* to enter into a situation (Kemper, 1981; Lavega et al., 2018). On the one hand, these were emotional states that showed the levels of activation experienced by the castellers when they are deeply committed to overcoming the relational and emotional challenge of building a human tower. On the other hand, tension and nerves were also associated with fear of the uncertainty of the outcome of their intervention or of possible damage to their physical integrity. At the same time, when the objective was not achieved, it was easy to identify expressions of emotional discomfort associated with sadness or anger. In general, a predominance of well-being corresponding to the emotion of joy was observed in the different phases of the construction of HT.

#### The Preparation in the Square. When Performing in Your Square Intensifies Emotions

Upon reaching the square, each *casteller* knows where to go; as pieces of a great cooperative puzzle, each person places themselves in their rightful place. The *castellers* of the *pinya* and the *tronc* look for their companions who share the same position in the HT. The *canalla*^8^ team goes to a room inside the City Hall, there, in a relaxed atmosphere, they are protected from the emotional pressure that comes from being in the square. The children will go out to act just as they are called. At the same time, the staff of the HT (*caps de colla*), meets, within the City Hall, to organize and define some strategic rules for the performance of that day; players called *cap de pinya*^9^ and *cap de tronc*^10^ stand in the square in order to check if all the *castellers* of the respective positions are there.

The players take advantage of these moments to initiate a very important cooperative action, putting on their sashes with the help of another *casteller*. This cooperative action is a testimony to an important casteller ritual. The sash symbolizes strength to withstand the physical effort; cooperation, as it will allow the castellers to ascend it; and identity, as it has traditionally been a very important element in traditional Catalan culture (Costes and Lavega-Burgués, 2019).

When you arrive at the square you look for your companions, we the players of the same position usually met each other. Then we start the ritual of wearing the sash. The sash has to fit well, very well-adjusted, because it's a very important element. You have to feel good! (E.7)

The *grallers*^11^ and *tabalers*^12^ are usually located on one side of the square. Finally, the spectators take up the free spaces of the square.

This preparation of the HT is a context of maximum social intensity (contact with colleagues, with *castellers* from other *collas*, with the public). This extraordinary social energy also originates an emotional energy (Collins, 2009), where pressure and nerves confirm this intense social-emotional meaning for the *castellers*.

Building a Human Tower in Paeria Square is always complicated. This is complicated by the pressure we create for ourselves. (E.4)

Nerves, we feel pure nerves. We are very nervous because it is our square.(E.10)

#### The Pinya. Transforming Nerves into Cooperative Efforts

The construction of the HT begins with the placement of the members of the *pinya*. As with any playful activity, each game, each performance is associated with a different experience, the result of which is unknown a priori. This circumstance, combined with acting in the square, causes nerves that each person experiences differently.

At first I get nervous because I want it to go well. These are healthy nerves, in other words, they are nerves that cheer you up. You feel the nerves in your stomach, and as if it were a voice that encourages you: go! go! (E. 9).

At that moment, when the first pieces of that big puzzle start to fit together, the *castellers* who make up the *tronc* also tell of experiencing nerves. Some of them explain strategies that help them to relax while the *piny*a is being closed.

… the *pinya* is almost finished, and you are very nervous. And you go looking for the Human Tower to be well prepared. You talk and ask, do you see it well, do you see it well? Yes of course! (E.8)

In the place where I am, I feel nervous, and I try to get relaxed. Looking ahead, I analyse if I see that the Human Tower is well prepared. This action relaxes me because I feel so much nervousness. (E.5)

At this phase, the statistical technique of the CRT shows a predominance of comments expressing states of discomfort in comparison with welfare stories.

At this point, the *castellers* of the *pinya* emphasize the importance of relationships with other colleagues. It helps a lot to know with whom you are going to cooperate directly, to generate the necessary confidence to do a good team work. It is very important that all the *castellers* feel comfortable. It is a good time to talk, greet each other, agree on small movements to ensure the position and feel that everyone is comfortable. All of this will be merged and will transform the motor action into emotional states.

When we go to the Human Tower, the names of the companions are indicated, and they are assigned to different positions at the *pinya* floor. in those moments each person observes who is on both sides; David is on the right, Ramón on the left. If they are the same ones you train with, you have confidence, it is an added positive element for the Human Tower. (E.7)

I always try to observe, to see if I receive a new partner, I try to know his name in case I need to ask him for something. Also, we all look at each other, to confirm that we are all well placed and comfortable. If it is necessary to take a step to the right, we talk to each other and we do it together. sometimes we shake hands depending on who, especially the first laces, is cool, there are looks, there is a very nice feeling (E.9)

#### The tronc. A Position to Activate the Emotional Flow

The *tronc* is the second cooperative network of a HT. The comments are oriented in the opposite direction to the *pinya* phase, as confirmed by the classification tree. The narratives show that the *castellers* of the *tronc* live their function intensely, with concentration, with pleasure and even disconnecting from the outside environment, that is, activating the sensation of flow (Csikszentmihalyi, 2014).

It's like a total disconnection. Sometimes I notice that we have built a human tower and when it is finished I don't remember any comments or anything that has happened. I have disconnected, I have been centred on the HT. (E.8)

The different floors are part of the same system of a cooperative motor communications network (Parlebas, 2001). Hence, while the *castellers* of the *tronc* express well-being, the *pinya* expresses feelings of nervousness as the companions of the *tronc* go up and build the HT.

You feel a lot of nerves, because as the HT is moving as the companions go up. You notice how goes up. and then you disconnect or think that the HT falls. (E.10)

*Castellers* put themselves to the test to overcome the physical risk (Collard, 2002, 2014), associated with the possible damage that can be done by *castellers* if the HT falls down. This may be due to a person getting on or off the HT, or because the HT is not consistent enough. Sometimes a *casteller* has not been able to withstand such physical effort. It is then that energy, decision, relationship and emotion become intertwined (Lavega-Burgués et al., 2020).

The sense of responsibility of *castellers* leads them to worry about the negative consequences (pain) that they may cause in the members of the community.

When you see that something is wrong, the first thing you think about is that it doesn't end up worse. So for me it is to protect the *crossas*, to hold the HT, in order the HT doesn't beat me because there are many people there holding and each one has to do his job. I have to hold there as long as I can in that moment so that it doesn't fall down because I can't hold… then you have to hold it. (E.7)

#### The Human Tower Is Crowned. The Effervescence of Collective Emotions

It is at this time, when the HT is about to be loaded, that the *castellers* experience the most nerves. When the HT is crowned and the *enxaneta*^13^ raises its arm, there is an effervescence of collective emotions. At that moment the sound of the *grallas* and *tabals*^14^ announces the coronation, so that all the *castellers* join in the emotional euphoria. The communion with the audience is maximum and they confirm the collective joy with applauses. The emotional states of previous moments are transformed: tension is replaced by joy, which is often accompanied by a need to cry.

It is very curious, at that moment when the HT is loaded, the audience applauds, and for me it is the moment of greatest nervousness. And when the audience applauds you feel a “UFF” (E.5)

When you hear the fin and you see the reaction of the people, it's like Barça has scored a goal, it's brutal, everyone applauding, it's very, very nice. (E.9)

When the HT l is crowned, everything is emotion. Sometimes I would cry, I would cry! It is an emotion that explains everything we have achieved; it also shows that the effort we are making is worthwhile. (E.6)

#### Going Down the Human Tower. A Network of Networks that Makes Well-Being Emerge

Then, it is time to go down the HT. This action causes an intense state of well-being in all *castellers*, regardless of the position they occupy. It is the result of a great physical, relational effort that finds a translation into intense positive emotions. All this generates a state of collective well-being, which is underlined by all the informants.

And when you start to go down, you start to relax and when you download, it is the joint joy of everyone because yes those who go up are the ones that are seen, but really the HT We all do it, it's a lot of joy, I don't know. (E.5)

### The External Logic of the Human Towers. A Context that Activates Collective Emotions

The analysis of the comments identifies two key emotional units of a HT performance: the collective movement from the *colla* to the square (beginning of the ritual) and the collective celebration after the construction of the HT.

#### From the Social Building to the Square. The Cooperative Casteller Emotional Contagion Begins

The emotional contagion (Collins, 2009) in the *Castellers* community begins from the same moment in which all together move from the social building to the square. The *grallas* and *tabals* are at the forefront of the group, leading this festive emotional contagion with their songs. The children go in a group, with the rogue team. The other people move through affinity groups that are normally related to the parts of a HT or by some family relationship.

On the day of the performance, we have the ritual of coming to the venue. Here we meet and all go down together, that is, in parades to the Paeria Square. We go down the main street and well, it is to walk around your house, through your city, it is all a pride, let's go! (E.7)

This study confirms the Lagardera et al. (2018, p. 35) findings in identifying the emotional map of semantic units of emotions elicited in cooperative games with competition. Emotional well-being and discomfort are activated by key factors as cooperation, the emotional climate of the group, the efficiency of cooperation and the uncertainty of the outcome of the performance. Therefore, we can state in accordance with the CRT (node 2) well-being and certain discomfort are an inseparable binomial: relaxed comments, applause, chants, greetings to family and friends trigger the first emotions of the day: joy, humor, satisfaction, security. At the same time, negative emotions also appear: nerves, anxiety, intrigue, insecurity, fear of possible falls.

The day you perform at home, since the moment you wake up you know you're going to perform in the Paeria. I love to move around with the kids. I love that they see us, I love that they live the party. the truth is that everything is really nice. (E.9)

I usually talk to my mother, my sister, I talk to people to keep myself distracted. Besides, I don't want to think, because there in Paeria Square there have been many falls. children have fallen down many times. (E.10)

#### Celebration. The Ecstasy of Cooperative Well-Being

At the end of a construction, when the collective celebration is made, as the results show (node 1, CRT), there is an intense predominance of the emotional state of well-being.

At that moment we want to celebrate it. We celebrate that moment by singing, jumping, hugging, kissing. We can do whatever it takes (laughs), yes. (E.10)

Yes, in that moment you embrace with everybody, it doesn't matter who it is. it's the joint joy of everybody because yes those who go up are the ones who are seen, but really the Human Tower is made by all of us and you embrace with everybody and you feel a lot of joy. (E.5)

Regardless of the position of the *castellers*, all the *castellers* are united by the joy they share with their companions.

## Conclusions

The emotional states in the Human Towers (HT) are the result of all the interpersonal experiences that take place before, during and after building a HT. All this, in a unitary and intertwined way, are different dimensions of the same festive phenomenon characterized by relational and emotional contagion, that is, the *casteller* ritual of a performance in the square.

This study contributes to a better understanding of the HT' s condition as an intangible cultural heritage of Catalonia. The emotional contagion confirms the sense of identity and cultural belonging that HT transmits in the protagonists and also in the spectators of the *casteller* phenomenon.

The statistical analysis of the quantitative data combined with the qualitative analysis of the narrative data allows us to understand the emotional states in the *casteller* context. The nature of the emotions is conditioned by the nature of the social situation in which these emotions are experienced. According to Kemper (1981) the biological nature of emotions is accompanied by the social nature of the situation, so that a necessary link is identified between affective subjectivity (the actor) and objective social situation (in our case, the HT).

The statistical technique of CRT shows the union of semantic units of internal logic and external logic in the same node, that is, they trigger the same emotional experience. This finding confirms the ethnomotor condition of the HT (Parlebas, 2001), where both the motor building of the HT (internal logic) and the relational exchanges in other contexts (external logic) are dimensions of the same socio-emotional phenomenon.

With a view to future research, it would be interesting to know whether these tendencies in emotional states are repeated in other contexts where HT are built, that is, in performances in squares in other cities and in the rehearsals in the place of the *colla*.

## Data Availability Statement

The raw data supporting the conclusions of this article will be made available by the authors, without undue reservation.

## Ethics Statement

The studies involving human participants were reviewed and approved by Ethics Committee for Clinical Research of the Catalan Sports Council. The patients/participants provided their written informed consent to participate in this study.

## Author Contributions

SD-S, CF, and PL-B: substantial contribution to study conception and design and preparation and participation in the empirical work. SD-S, CF, QP, RL-P, and PL-B: preparation of the document for approval by the ethics committee. SD-S, CF, QP, RL-P, AR-A, US, AC, and PL-B: database revision. SD-S, CF, QP, RL-P, MP, and PL-B: discussion of data analysis strategies. SD-S, CF, MP, QP, RL-P, AR-A, US, AC, and PL-B: writing of the manuscript. All authors: contributed to the article and approved the submitted version.

## Conflict of Interest

The authors declare that the research was conducted in the absence of any commercial or financial relationships that could be construed as a potential conflict of interest. The reviewer MS declared a shared affiliation, with no collaboration, with several of the authors US and AR-A to the handling editor at the time of the review.

## Footnotes

^1^*Gralla(s)*: a popular wooden musical instrument with a truncated cone shape that produces sound by blowing through the reed.

^2^*Casteller(s) - a(s*): person who participates in the construction of Human Towers and who belongs to a group.

^3^*Pinya*: part of the human tower formed by a group of people who make the base of a human tower, touching the feet to the ground.

^4^*Tronc*: part of the human tower that comprises from the ground floor that is below the *segons* to the floor immediately before the *dosos*, and which outlines the structure of the Human Tower vertically.

^5^*Pom de dalt*: Part of the human tower made up of the *dosos* floor, the *aixecador* floor and the *enxaneta* floor.

^6^*Colla:* Organized group of *castellers* under the same name, the same shirt colour and led by a *cap de colla*.

^7^*Cap de colla*: person who commands and directs the technical work of all the *castellers* of a *colla*.

^8^*Canalla*: group of the youngest *castellers* of the *colla*, formed by les *enxanetes, aixecadors* and *dosos*.

^9^*Cap de pinya*: person who directs the technical work of the *castellers* de la *pinya*.

^10^*Cap de tronc*: person who directs the technical work of the *castellers* del *tronc*.

^11^*Graller(s)*: person who plays the *gralla* instrument.

^12^*Tabaler(s)*: person who plays the *tabal* instrument.

^13^*Enxaneta*: child crowning a Human Tower.

^14^*Tabal(s): percussion instrument that consists of a cylindrical wooden box where the bases are two tight skins*.
